# Chiropractic treatment of older adults with neck pain with or without headache or dizziness: analysis of 288 Australian chiropractors’ self-reported views

**DOI:** 10.1186/s12998-019-0288-1

**Published:** 2019-12-18

**Authors:** Dein Vindigni, Laura Zark, Tobias Sundberg, Matthew Leach, Jon Adams, Michael F. Azari

**Affiliations:** 1 Chiropractic Discipline, School of Health and Biomedical Sciences, RMIT University, Melbourne, Australia; 2 School of Psychology, Faculty of Health, Deakin University, Burwood, Melbourne, Australia; 3 Musculoskeletal & Sports Injury Epidemiology Center (MUSIC), Institute of Environmental Medicine, Karolinska Institute, Stockholm, Sweden; 4 Australian Research Centre in Complementary and Integrative Medicine (ARCCIM), Faculty of Health, University of Technology Sydney, Sydney, Australia; 5 Department of Rural Health, University of South Australia, Adelaide, Australia; 6 Private practice, Azari Chiropractics, Mount Waverley, Melbourne, Australia

**Keywords:** Chiropractic, Headache, Older adults, Neck pain, Survey

## Abstract

**Background:**

Neck pain is a leading cause of individual and societal burden worldwide, affecting an estimated 1 in 5 people aged 70 years and older. The nature and outcomes of chiropractic care for older adults with neck pain, particularly those with co-morbid headaches, remains poorly understood. Therefore, we sought to ascertain: What proportion of Australian chiropractors’ caseload comprises older adults with neck pain (with or without headache); How are these conditions treated; What are the reported outcomes?

**Methods:**

An online survey examining practitioner and practice characteristics, clinical patient presentations, chiropractic treatment methods and outcomes, and other health service use, was distributed to a random nationally representative sample of 800 Australian chiropractors. Quantitative methods were used to analyze the data.

**Results:**

Two hundred eighty-eight chiropractors (response rate = 36%) completed the survey between August and November 2017. Approximately one-third (M 28.5%, SD 14.2) of the chiropractors’ patients were older adults (i.e. aged ≥65 years), of which 45.5% (SD 20.6) presented with neck pain and 31.3% (SD 20.3) had co-morbid headache. Chiropractors reported to combine a range of physical and manual therapy treatments, exercises and self-management practices in their care of these patients particularly: manipulation of the thoracic spine (82.0%); activator adjustment of the neck (77.3%); and massage of the neck (76.5%). The average number of visits required to resolve headache symptoms was reported to be highest among those with migraine (M 11.2, SD 8.8). The majority of chiropractors (57.3%) reported a moderate response to treatment in reported dizziness amongst older adults with neck pain. Approximately 82% of older adult patients were estimated to use at least one other health service concurrently to chiropractic care to manage their neck pain.

**Conclusion:**

This is the first known study to investigate chiropractic care of older adults living with neck pain. Chiropractors report using well-established conservative techniques to manage neck pain in older adults. Our findings also indicate that this target group of patients may frequently integrate chiropractic care with other health services in order to manage their neck pain. Further research should provide in-depth investigation of older patients’ experience and other patient-reported outcomes of chiropractic treatment.

## Background

Neck pain, with or without headache or dizziness, is a leading cause of disability affecting over 350 million people worldwide as of 2015 [1, 2]. The prevalence of neck pain is also high among older people. In Denmark, for instance, the one-month prevalence of neck pain is reported to be as high as 22% amongst those aged 70 years and over [3]. Neck pain causes a substantial economic burden, attributable to healthcare and insurance costs, decreased work productivity, and work absenteeism [4]. Furthermore, it is associated with myriad other health problems [5], prominently co-morbid headaches [6–8], and decreased health-related quality of life [9].

Neck pain in older adults is not extensively studied and this population is generally poorly represented in musculoskeletal research [10–12]. Many older adults are restricted in their pain medication use due to multiple co-morbidities and associated polypharmacy, and/or increased risk of harmful side effects due to age-related pharmacokinetic and pharmacodynamic changes [13, 14]. Therefore, non-pharmacological treatments including chiropractic methods may be a particularly valuable service for older adults, who face increased barriers to pain relief [15].

Many older adults in Australia suffering from neck pain and associated conditions seek chiropractic treatment [16]. In fact, musculoskeletal complaints, most commonly back and neck pain, account for the vast majority of chiropractic consultations in Australia [16–20]. According to the latest survey of Australian chiropractic clinical activity, 93.6% of Chiropractors stated that neck pain was a common presenting complaint in their practices [16]. However, relatively little is known about the nature and effectiveness of chiropractic treatment for neck pain in older adults. This is particularly so in Australia. As ‘chiropractic care’ encompasses numerous and wide-ranging treatment modalities, information about the specific methods used and their outcomes, is critical to understanding what constitutes usual contemporary chiropractic care for older adults with neck pain.

Previous national surveys of Australian chiropractors have predominantly focused on workforce characteristics. While providing valuable demographic insights – namely, a predominance of male, 25–49 year old, university-educated chiropractors working full-time in major cities [21–23] – these surveys have largely overlooked clinical management characteristics. Adams and colleagues [16] have begun to address this gap by surveying a nationally representative sample of Australian chiropractors on not only workforce characteristics, but characteristics of their clinical care. Their results showed that the manipulative methods most commonly employed by chiropractors were high velocity, low amplitude adjustment/manipulation/mobilisation (82.2%), followed by extremity manipulation (58.5%), drop-piece techniques (53.7%) and instrument-aided manipulation (52.3%). The most commonly employed *musculoskeletal* interventions were soft tissue therapy, trigger point therapy, massage therapy and/or stretching (66.1%), followed by specific exercise therapy/rehabilitation/injury taping (49.3%) and heat/cryotherapy (16.6%). However, considerable knowledge gaps in the field still remain. For instance, despite being a major clinical concern, clinical management characteristics for neck pain remain largely unexplored. Furthermore, prior investigations of Australian chiropractic care have given little consideration to the older adult patient population. Given the ageing population and the consequent increase in the prevalence of neck pain projected over the coming decades [4], there is a need to better understand the chiropractic interventions used to treat older adults with neck pain.

In direct response to this research gap, we surveyed a large representative sample of practicing Australian chiropractors about their treatment of neck pain and co-morbid headaches in older adult patients aged ≥65 years. Specifically, the study aimed to examine: a) the demand for chiropractic services among older patients with or without headache [expressed as the proportion of patient-load]; b) how neck pain (with or without headache) is treated by chiropractors; and c) the reported outcomes of these treatments.

## Methods

### Design

The study was a national, cross-sectional survey of Australian chiropractors and constituted a sub-study of the Australian Chiropractic Research Network (ACORN) Project [24–26]. The survey instrument appears in the Appendix. The ACORN Project has been described in detail elsewhere, but in short, it is the largest voluntary practice-based research network (PBRN) in the world exclusively focused on chiropractic practice [26]. ACORN is designed and administered by senior researchers at the Faculty of Health, University of Technology Sydney (UTS), received ethics approval from the UTS Human Research Ethics Committee (Approval number: 2014000027) and has been officially registered as an international practice-based research network with the Agency for Healthcare Research and Quality PBRN Resource Centre since 2015 [25].

### Participants & recruitment

Participants for the study were recruited from the ACORN database of practicing Australian chiropractors, which consisted of 1680 chiropractors. The sample was nationally representative of chiropractors in Australia in terms of key indicators such as age, gender and practice location [24, 26]. A sample size of 400 was calculated by an experienced biostatistician to ensure generalizability of the data. We doubled this sample size to ensure sufficient participation. Therefore, a random sample of 800 chiropractors was selected from the ACORN database and invited to participate by email (between August and November 2017), which included a link to the online survey. All participants were provided with a detailed participant information sheet. Participation was voluntary and consent was given by participants’ opting in to the survey. All information was kept confidential on university computer servers, anonymized and pooled before reporting. Three follow-up reminder emails were sent to participants approximately 2 weeks apart.

### Survey

In addition to demographic information, the survey aimed to collect data on the reported treatment of older adults with neck pain with/out headache by Australian chiropractors, as well as the practicing chiropractors’ perception of the effectiveness of such treatments. The online survey was developed by MFA, drawing from the relevant literature and refined in consultation with the co-authors of this manuscript. The survey was piloted by three practicing Australian chiropractors, the results of which led to minor changes in the wording of some questions. The survey instrument was created and distributed using Qualtrics software. The survey comprised of 28 questions (12 MCQs, and 14 Likert-scale items, and 2 open-ended questions) addressing: chiropractors’ demographic and practice characteristics; estimates of how often the chiropractors treat older adults with neck pain (with and without headaches); the treatment methods employed for this population; estimates of treatment effectiveness; and estimates of usage of other health services among this population. The average completion time of the survey was 5–6 min.

### Analyses

Descriptive statistics were used to report frequencies and relative proportions for categorical variables, and means and standard deviations (*SD*) for continuous variables. Pearson’s chi-square (χ^2^) tests were performed to test for associations between categorical variables, and independent samples *t*-tests and ANOVAs were performed to test for group differences in continuous variables. Where expected frequencies were too small (i.e. < 5), categories were collapsed into broader groupings for χ^2^ tests. Outliers, defined as values with a standardised residual greater than ±3.29, were winsorized (i.e., replaced with the next highest observed value that wasn’t an outlier [27]). Observations with missing data were excluded from the analyses. Unless otherwise stated, results were considered significant if *p* < .05. Data were analysed using IBM SPSS Statistics version 22.

### Ethics

In addition to the ACORN ethics approval, the study reported here was reviewed and approved by a College Human Ethics Advisory Network (CHEAN) subcommittee of RMIT University Human Research Ethics Committee (HREC) (Approval number: SEHAPP 23–17).

## Results

Two hundred and eighty-eight chiropractors completed the survey, yielding a response rate of 36%.

### Demographic characteristics

Two hundred and seven participants were male and 81 were female (Table 1). More than 55% of respondents were between 45 and 64 years in age and had been in practice for more than 20 years. Approximately 54% of them lived in New South Wales or Victoria and approximately 46% practiced in the outer suburban settings (Table 1). It is important to note that over 80% of the respondents were either in solo practice or in practiced with other chiropractors. While over 47% of them stated that they had more than 100 patient visits per week, 88% of the respondents reported to have less than 9 new patients per week (Table 1).

**Table 1:** Demographic and Practice Characteristics reported by Chiropractors (*N* = 288)

	*n* (%)
Gender	
Male	207 (71.88)
Female	81 (28.13)
Age (years)	
25–34	40 (13.89)
35–44	68 (23.61)
45–54	91 (31.60)
55–64	75 (26.04)
≥ 65	14 (4.86)
Years in practice	
0–5	25 (8.68)
6–10	32 (11.11)
11–15	42 (14.58)
16–19	28 (9.72)
≥ 20	161 (55.90)
State/territory of practice	
New South Wales	91 (31.60)
Victoria	66 (22.92)
Western Australia	49 (17.01)
Queensland	37 (12.85)
South Australia	32 (11.11)
Australian Capital Territory	9 (3.13)
Northern Territory	2 (0.69)
Tasmania	2 (0.69)
Main practice location	
suburb	132 (45.83)
Inner city	63 (21.88)
Regional centre	63 (21.88)
Rural	30 (10.42)
Main practice type	
Private practice – Solo	122 (42.36)
Private practice – Group of chiropractors	111 (38.54)
Private practice – Multidisciplinary (excluding GPs)	49 (17.01)
Private practice – Multidisciplinary (including GPs)	6 (2.08)
Patient visits in practice per week	
< 30	24 (8.33)
31–60	60 (20.83)
61–100	67 (23.26)
> 100	137 (47.57)
New patients in practice per week	
0–3	123 (42.71)
4–8	131 (45.49)
9–20	27 (9.38)
> 20	7 (2.43)

### Clinical presentations

On average, chiropractors reported that 28.5% (*SD* = 14.2) of their patients were older adults. This proportion was slightly higher among practitioners working in rural/regional locations (*M* = 30.9%, *SD* = 14.5) than those working in urban locations (*M* = 27.4%, *SD* = 13.9), though this difference just missed statistical significance (*t* (286) = − 1.95, *p* = .052). The proportion of older adult patients was not reported to differ according to the practitioner’s gender (*t* (286) = 0.28, *p* = .781), age group (*F*(4, 283) = 0.71, *p* = .587), or number of years in practice (Welch’s *F* (4, 72.90) = 0.58, *p* = .679).

The respondents also reported, on average, that almost half of their older adult patients had presented with neck pain (*M* = 45.5%, *SD* = 20.6). This was found to differ according to the practitioner’s gender (*t*(262) = − 2.82, *p* = .005) and age group (Welch’s *F*(4, 75.22) = 3.00, *p* = .024). Specifically, this estimated proportion of older adult patients with neck pain was significantly higher among male (*M* = 47.7%, *SD* = 19.7) than female (*M* = 39.8%, *SD* = 22.0) practitioners and, according to Games-Howell post hoc tests, was significantly higher among practitioners aged 25–34 years (*M* = 51.5%, *SD* = 17.49) than those aged 65+ years (*M* = 35.0%, *SD* = 14.70) (*M*_difference_ = 16.50, *p* = .016). However, the estimated proportion of older adult patients with neck pain did not differ according to the number of years the chiropractor had been in practice (*F*(4, 259) = 1.18, *p* = .319).

The surveyed chiropractors reported that approximately one third of their older adult patients with neck pain had also presented with headache (*M* = 31.3%, *SD* = 20.3). Of these patients, 36.5% (*SD* = 23.1) reportedly had cervicogenic headache, 24.0% (*SD* = 18.0) had tension-type headache, and 8.5% (*SD* = 7.6) had migraine headache. Congruently, the majority (79.6%) of chiropractors reported that cervicogenic headache was the most common type of headache experienced by their older adult patients with neck pain (see Table 2).

**Table 2:** Results of Categorical Survey Questions on Clinical Presentation, Treatment Methods, Treatment Outcome and Other Service Use

	*n* (%)
Most common headache in older adult patients with neck pain^a^	
Cervicogenic headache	210 (79.55)
Tension-type headache	49 (18.56)
Medication overuse headache	3 (1.14)
Migraine	1 (0.38)
Treatment/s typically used to treat older adult patients with neck pain^b,c^	
Adjustment/manipulation of thoracic spine	209 (81.96)
Activator adjustment of neck	197 (77.25)
Massage of neck (including trigger point therapy, Nimmo)	195 (76.47)
Manual neck adjustment/manipulation	173 (67.84)
Mobilisation of neck	164 (64.31)
Stretching exercises	149 (58.43)
Passive stretching of neck (including traction)	141 (55.29)
Heat/cold application	78 (30.59)
Other	75 (29.41)
Drop-piece (Thompson) adjustments of neck	47 (18.43)
Over-the-counter analgesics and/or anti-inflammatories	31 (12.16)
Upper cervical toggle recoil adjustment	31 (12.16)
Ultrasound/Interferential	13 (5.10)
Typical response of dizziness to treatment (in older adult patients with neck pain who also have dizziness)^b^	
No response	4 (1.57)
Mild	35 (13.73)
Moderate	146 (57.25)
Excellent	70 (27.45)
Services used by older adult patients with neck pain (in addition to chiropractic care)^b,c^	
Over-the-counter analgesics and/or anti-inflammatories	210 (82.35)
Massage (by a massage therapist)	168 (65.88)
Prescription medications (for pain or inflammation)	166 (65.10)
Physiotherapy	114 (44.71)
Acupuncture/Dry-needling	84 (32.94)
Other	15 (5.88)

^a^*N* = 264, ^b^*N* = 255, ^c^Categories are not independent (i.e., respondents could select as many options as applicable)

Beyond headaches, chiropractors reported, on average, that 28.0% (*SD* = 20.1) of their older adult patients with neck pain suffered from dizziness, with 24.1% (*SD* = 21.3) suffering from cervicogenic dizziness. On average, 44.2% (*SD* = 28.2) of older adult patients with neck pain were reportedly concerned about, or afraid of, falling.

### Treatment methods

The three most frequently used treatments for older adult patients presenting with neck pain, as reported by the surveyed chiropractors, were adjustment/manipulation of the thoracic spine (82.0%), activator adjustment of the neck (77.3%), and massage of the neck (76.5%) (see Table 2).

Most (85.9%) chiropractors reported to employ more than three different methods to treat the older adult neck pain patient population. This was the case for both male and female practitioners (χ^2^(1) = 0.73, *p* = .393). However, chiropractors under 45 years of age were significantly more likely to claim to use more than 3 treatments (93.3%) than those aged 45 years or older (81.8%) (χ^2^(1) = 6.37, *p* = .012). Additionally, the number of treatments reported to be used (≤3 vs. > 3) differed according to the chiropractor’s number of years in practice (χ^2^(2) = 7.14, *p* = .028) – the 97.9% of chiropractors who each had less than 10 years of chiropractic clinical experience claimed to use more than 3 treatments, compared to 85.2% of those with 11–19 years of clinical experience, and 82.3% of those with 20 or more years of clinical experience.

### Treatment outcomes

Chiropractors reported that the average number of chiropractic visits that older adult patients with neck pain received, before they considered their complaint to be resolved, was highest among those with migraine headache (*M* = 11.2, *SD* = 8.8), followed by those with cervicogenic headache (*M* = 9.3, *SD* = 6.3), tension-type headache (*M* = 9.3, *SD* = 5.9), and those without headache (*M* = 9.2, *SD* = 5.7).

The number of reported chiropractic visits did not significantly differ by the chiropractor’s gender for patients with cervicogenic headache (*t*(253) = − 0.32, *p* = .750), tension-type headache (*t*(253) = − 1.79, *p* = .075), or no headache (*t*(253) = − 1.57, *p* = .118). However, male chiropractors estimated a significantly higher number of visits (*M* = 12.04, *SD* = 8.62) than female chiropractors (*M* = 8.7, *SD* = 7.59) among their older adult patients with neck pain and concurrent migraine headache (*t*(253) = − 2.85, *p* = .005).

The number of chiropractic visits for older adult patients with neck pain was not reported to significantly differ by the practitioner’s age group, regardless of concurrent headache type (no headache: *F*(4, 250) = 0.33, *p* = .855; tension-type headache: *F*(4, 250) = 1.51, *p* = .199; migraine headache: *F*(4, 250) = 0.61, *p* = .654; cervicogenic headache: *F*(4, 250) = 1.72, *p* = .146). Likewise, the number of chiropractic visits for older adult patients with neck pain was not claimed to significantly differ by number of years in practice (no headache: *F*(4, 250) = 0.76, *p* = .553; tension-type headache: *F*(4, 250) = 1.24, *p* = .293; migraine headache: Welch’s *F*(4, 69.32) = 1.01, *p* = .407; cervicogenic headache: *F*(4, 250) = 0.96, *p* = .430).

With regard to older adult patients with neck pain who also had dizziness, most chiropractors rated the typical response of dizziness to their treatment as ‘moderate’ (57.3%) (compared to excellent, mild or no response) (see Table 2). The proportion of chiropractors who rated the treatment response of dizziness as excellent did not significantly differ according to the chiropractor’s gender (χ^2^(1) = 0.06, *p* = .805), age group (χ^2^(4) = 3.19, *p* = .527), or years in practice (χ^2^(4) = 2.21, *p* = .697).

### Other health services used by patients

On average, chiropractors reported that 37.9% (*SD* = 26.8) of their older adult patients with neck pain use other health services in addition to chiropractic care. These included (in descending order of frequency): over-the-counter analgesics and/or anti-inflammatories; massage (by a massage therapist); prescription medications (for pain or inflammation); physiotherapy; and acupuncture/dry-needling (Table 2).

Female chiropractors reported a significantly higher rate of other health service use among their older adult patients with neck pain (*M =* 43.4%, *SD* = 28.5) compared to male chiropractors (*M =* 35.8%, *SD* = 25.9) (*t*(253) = 2.03, *p* = .044). This also differed according to the chiropractor’s age group (*F*(4, 250) = 3.23, *p* = .013) and years in practice (*F*(4, 250) = 3.52, *p* = .008). Tukey’s post hoc tests revealed that chiropractors aged 25–34 years reported a significantly higher rate of other health service use among their older adult patients with neck pain (*M* = 47.7%, *SD =* 26.5) than those aged 65+ years (*M =* 21.2%, *SD =* 23.1) (*p* = .014), and chiropractors with 6–10 years of experience reported a significantly higher rate of other health service use (*M* = 53.1%, *SD* = 24.2) than those with 20+ years of experience (*M =* 34.6%, *SD* = 26.39) (*p* = .012).

## Discussion

To our knowledge, this is the first study to investigate the nature and perceived outcomes of chiropractic care of older adults living with neck pain with or without headache or dizziness.

Almost one-third of the chiropractors’ estimated case load represented older adults, and this was not dependent on the practice location or chiropractors’ age, gender, or number of years in practice. As expected, neck pain was reported to be common among older people who presented to chiropractors. In fact, neck pain was estimated to account for approximately half of the presenting complaints in this population. While we found statistically significant gender and age differences such that male practitioners in the 25–34 age group seemed to see more cases of neck pain in older adults, these differences are unlikely to be clinically meaningful. Together, these reported findings demonstrate significant demand for chiropractic services among older people with neck pain. They also have important implications for chiropractic education and research, as this is a population that the chiropractic profession serves to a substantial degree.

Chiropractors reported that among their older adult patients with neck pain, approximately one-third also presented with headache; this was estimated to be either cervicogenic (one-third), or tension-type (one-quarter) or migraine (less than 9%). This is consistent with the reported prevalence of tension-type headache, cervicogenic headache and migraine among older adults [28]. It is also important to note that respondents reported that over 44% of their older adult patients with neck pain were at risk of falls, and over one-quarter of them suffered from cervicogenic dizziness. This highlights an important area of disease prevention and health promotion in which the chiropractic profession may need greater educational and practice focus. This need has also been previously highlighted by chiropractic researchers in New Zealand [29]. Indeed, there is some preliminary evidence that manual therapy may be beneficial for improving balance and potentially reducing falls risk [30]. Future studies may consider investigating possible differences in the prevalence of falls or falls-risk between older people who routinely receive manual therapy (perhaps in addition to exercise) for their neck pain and those who do not.

Australian chiropractors reported to use diverse manual and physical therapy techniques to manage neck pain in older adults, including manipulation, mobilization, a wide range of exercises and self-management strategies. The most frequently reported treatments (reported by more than one-half of surveyed chiropractors), which may be viewed as constituting ‘usual chiropractic care’ for this condition in this population, were thoracic manipulation, massage manipulation and mobilization of the neck (including Activator adjustments), passive stretching and stretching exercises. These treatments reflect best practice in the use of manual therapy for neck pain in adults [31]. However, we note that almost 45% of the chiropractors surveyed did not report the use of exercise to manage neck pain with or without headaches in the elderly (Table 2). The evidence for the effectiveness of exercise is clear for these conditions and we recommend that more chiropractors follow current guidelines that recommend exercise therapy [32, 33].

The finding that younger (< 45 years of age) chiropractors (who presumably also account for those with less than 10 years of clinical experience) claimed to use more than three treatment modalities to manage neck pain in older adults is interesting. This may reflect limited clinical expertise, or on the other hand, a greater willingness to experiment with combinations of treatment modalities to achieve better results. Alternatively, it may be indicative of a greater willingness on the part of younger chiropractors to follow current clinical guidelines that recommend multimodal care [33] and hence the use of more treatment modalities. Further research is needed to explore these and other possible interpretations.

According to responses from the surveyed chiropractors, older adult patients with neck pain requiring the lowest dose of chiropractic treatment were generally without headache. As expected, the response of headaches to chiropractic treatment in older adults with neck pain was reported to vary according to headache type. Older adult patients with neck pain and tension-type headache, followed by cervicogenic and migraine headache, were reported to need progressively higher doses of treatment before they considered their condition to be resolved. However, many chiropractors justifiably commented that these presenting complaints rarely permanently ‘resolved’ in older adults and they needed long-term management. However, an intriguing finding was that male chiropractors estimated 50% more treatment visits were needed for older adult patients with neck pain and migraine headaches. It is important for future research to identify whether male chiropractors provide more treatment sessions to this subpopulation of older adults and to explore the reason/s for this.

Most chiropractors reported moderately positive responses (on a scale from no response to excellent response) to chiropractic treatments for dizziness in older adult patients with neck pain. This is a potentially important finding that could have significant ramifications for falls prevention strategies in primary care, as dizziness is an important risk factor for falls in older adults [34]. However, more rigorous research methodologies designed to test treatment effectiveness (i.e. randomised controlled trials) need to be employed in the future to investigate this possibility.

Interestingly, Australian chiropractors estimated that one in three older adult patients receiving chiropractic care for their neck pain also accessed other health services in order to manage their neck pain. These included (in descending order of frequency): over-the-counter analgesics and/or anti-inflammatories, massage (by a massage therapist), prescription medications (for pain or inflammation), physiotherapy, and acupuncture or dry-needling (Table 2). The reasons for this reported observation are not clear. One possible explanation is that neck pain in many older patients can be complicated by co-morbidities, meaning that these patients may be more resistant to chiropractic intervention. Further research investigating the patient perspective may help shed more light on this finding.

Of further interest was that female chiropractors’ estimates of the proportion of older adult patients with neck pain who simultaneously use other health services was significantly higher than their male colleagues. This was also the case for younger chiropractors and those with fewer years of clinical practice experience. Again, a number of possible explanations exist for these observations. It may be that the treatment approaches of female chiropractors are less clinically effective. It also may be that a female chiropractors’ relationship with their patient is more open, hence allowing their patients to more readily disclose the use of other health care services.

### Clinical implications

Given the high burden of illness associated with neck pain in older people, including those with comorbid headaches, and the societal burden worldwide, a better understanding of the nature and outcomes of chiropractic care may help inform improved, intra and multidisciplinary approaches to the management of this condition. This nationally representative study found that Australian chiropractors claim to employ commonly used manual and physical therapy techniques to manage neck pain in older people. Chiropractic care may therefore provide a particularly valuable service for older adults, particularly those unable to tolerate analgesic and anti-inflammatory medications.

Despite some variations in the volume and frequency of treatments reported between male and female chiropractors in this study, on average, the reported health-related outcomes for neck pain and associated comorbidities appear favourable for older adults. The findings also indicate that this target group of patients may frequently integrate chiropractic care with other health services in order to effectively manage and co-manage their neck pain. Providing evidence-based and patient-centred treatment options for older people experiencing neck pain and associated comorbidities may help facilitate improved pathways to healthy ageing in this population.

### Limitations and future research

This study used a self-reported questionnaire to gather data on the proportion of older Australian patients with neck pain (with or without headache) using chiropractic, the characteristics of chiropractors treating a higher or lower proportion of older people, how neck pain (with or without headache) was managed, and the outcomes of such treatments. Limitations of the study included: practitioner bias in the description of treatments; professional bias (i.e. having a favourable view of one’s own practices and their clinical effectiveness); and recall bias (as participants were required to respond to questions that relied on their memory, which can be considered inherently biased towards cases with favourable outcomes). Notwithstanding, it is difficult to gauge the influence of recall bias in this study as the respondents were not asked to produce estimations of their clinical encounters over a specified period of time. The large number of respondents, the national representativeness of the sample, and the strong patterns observed in the responses to questions represent important strengths of this study.

Future studies should aim to validate the findings of this survey, by surveying a large cross-section of older adult patients with neck pain, or by auditing a representative sample of the clinical files of chiropractors treating this condition. Further studies could also focus on exploring the differences between treatment patterns and outcomes between female and male chiropractors, other health-service use and predictors for improved treatment outcomes for neck pain (with or without the various types of headache).

## Conclusions

This is the first known study to investigate chiropractic care of older adults living with neck pain. The findings suggest that chiropractors use well-established manual and physical therapy techniques to manage neck pain in older adults. The favourable outcomes reported by participants highlight a potential role for using non-pharmacological multimodal therapeutic approaches for the management of neck pain in older adults. The findings also indicate that this target group of patients may frequently integrate chiropractic care with other health services in order to manage their neck pain. Understanding the patient’s motivation for using multiple services may shed light on the health care needs of this population. Further research should also explore how chiropractic treatment of neck pain in older adults impacts patient experience, and other patient-reported outcomes. Given the high prevalence of neck pain in older people, the evidence for the effectiveness of manual and physical treatments for neck pain, the reported demand for chiropractic care in this population, the barriers to pain relief, and concerns among older adults regarding polypharmacy, further studies are needed to provide a more solid evidence-base upon which clinical guidelines for chiropractic management and/or co-management of this condition can be developed. Until then, we recommend that the current clinical guidelines be followed.
